# Supplementary material for: On-Resin Selenopeptide Catalysts: Synthesis and Applications of Enzyme-Mimetic Reactions and Cyclization of Unsaturated Carboxylic Acids
Source: Molecules. 2025 Jan 22;30(3):480. doi: 10.3390/molecules30030480 (PMC11820528; doi:10.3390/molecules30030480)
Supplement: Supplementary file 1 [file molecules-30-00480-s001.zip › molecules-3422920-supplementary.pdf]

Supporting Information

Contents:

1. HPLC charts and MALDI-TOF-MS spectra for the peptides cleaved from resins **5a-g** and **6a-d** (Figure S1-S10). ········ p. S2-S11

2. Experimental data of the GPx-like peroxidase activity assay by the UV method (Figure S11). ········ p. S12

3. Experimental data of the GPx-like peroxidase activity assay by ^{1}H NMR (Figure S12). ········ p. S13

4. Molecular structures of H-UAKGEL-OH obtained by REMC/SAAP3D simulation and the subsequent clustering analysis (Figure S13). ········ p. S14

5. ^{1}H NMR spectra for cyclized products **8a**, **8b**, **8d**, and **8e** (Figure S14-S17). ········ p. S15-S16

6. Determination of the enantiomeric excess for cyclized product **8a** (Table S1). ········ p. S17

7. The second-order rate constants (k_2) for **5a-g** in comparison with those reported for other selenopeptides (Table S2). ········ p. S18

1. **HPLC charts and MALDI-TOF-MS spectra for the peptides cleaved from resins 5a-g and 6a-d.**

(a)

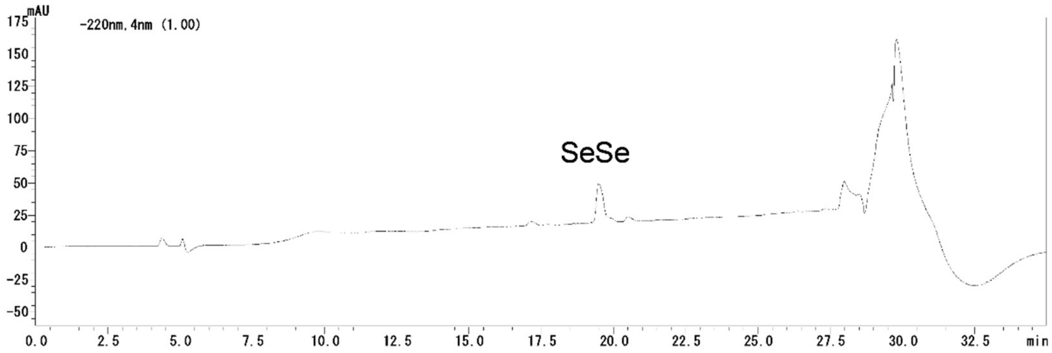

(b)

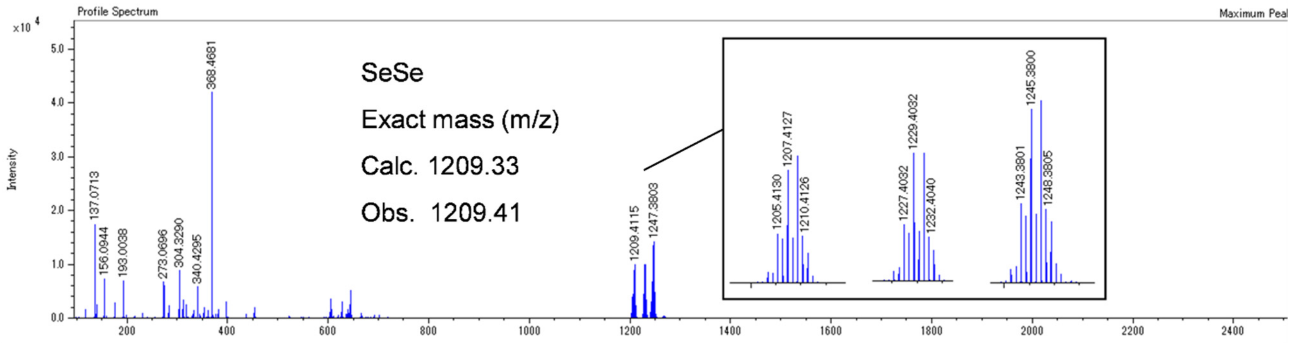

Figure S1. HPLC chart and MALDI-TOF-MS spectra for (H-UHGEL-OH)$_2$ cleaved from H-UHGEL-PAM (**5a**). (a) HPLC chart of the cleaved peptide mixture. (b) MALDI-TOF-MS spectrum for the SeSe peak.

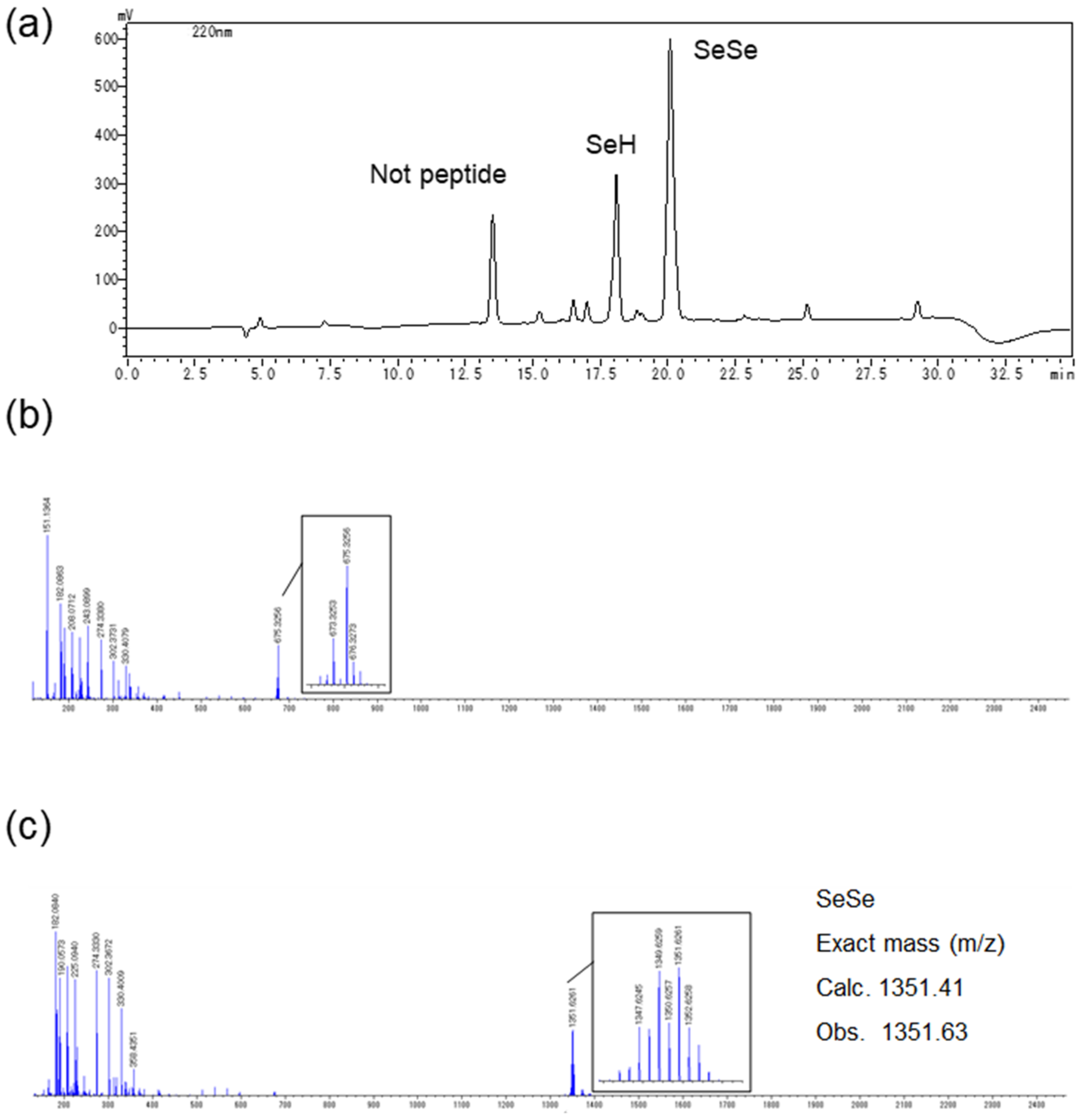

Figure S2. HPLC chart and MALDI-TOF-MS spectra for (H-UAHGEL-OH)$_2$ cleaved from H-UAHGEL-PAM (**5b**). (a) HPLC chart of the cleaved peptide mixture. (b) MALDI-TOF-MS spectrum for the SeH peak. (c) MALDI-TOF-MS spectrum for the SeSe peak.

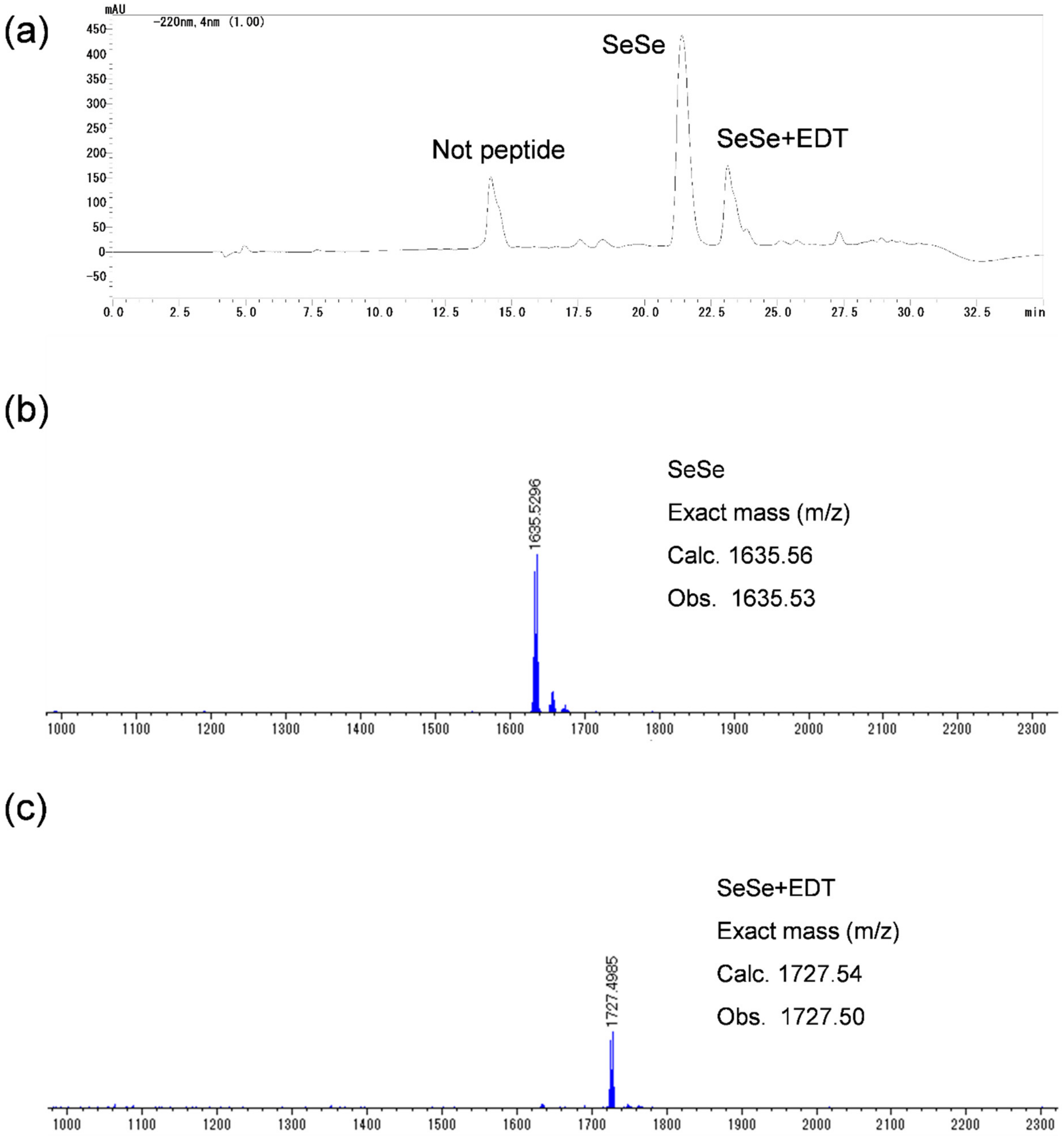

Figure S3. HPLC chart and MALDI-TOF-MS spectra for $(H\text{-}UAAAHGEL\text{-}OH)_2$ cleaved from H-UAAAHGEL-PAM (**5c**). (a) HPLC chart of the cleaved peptide mixture. (b) MALDI-TOF-MS spectrum for the SeSe peak. (c) MALDI-TOF-MS spectrum for the SeSe+EDT peak.

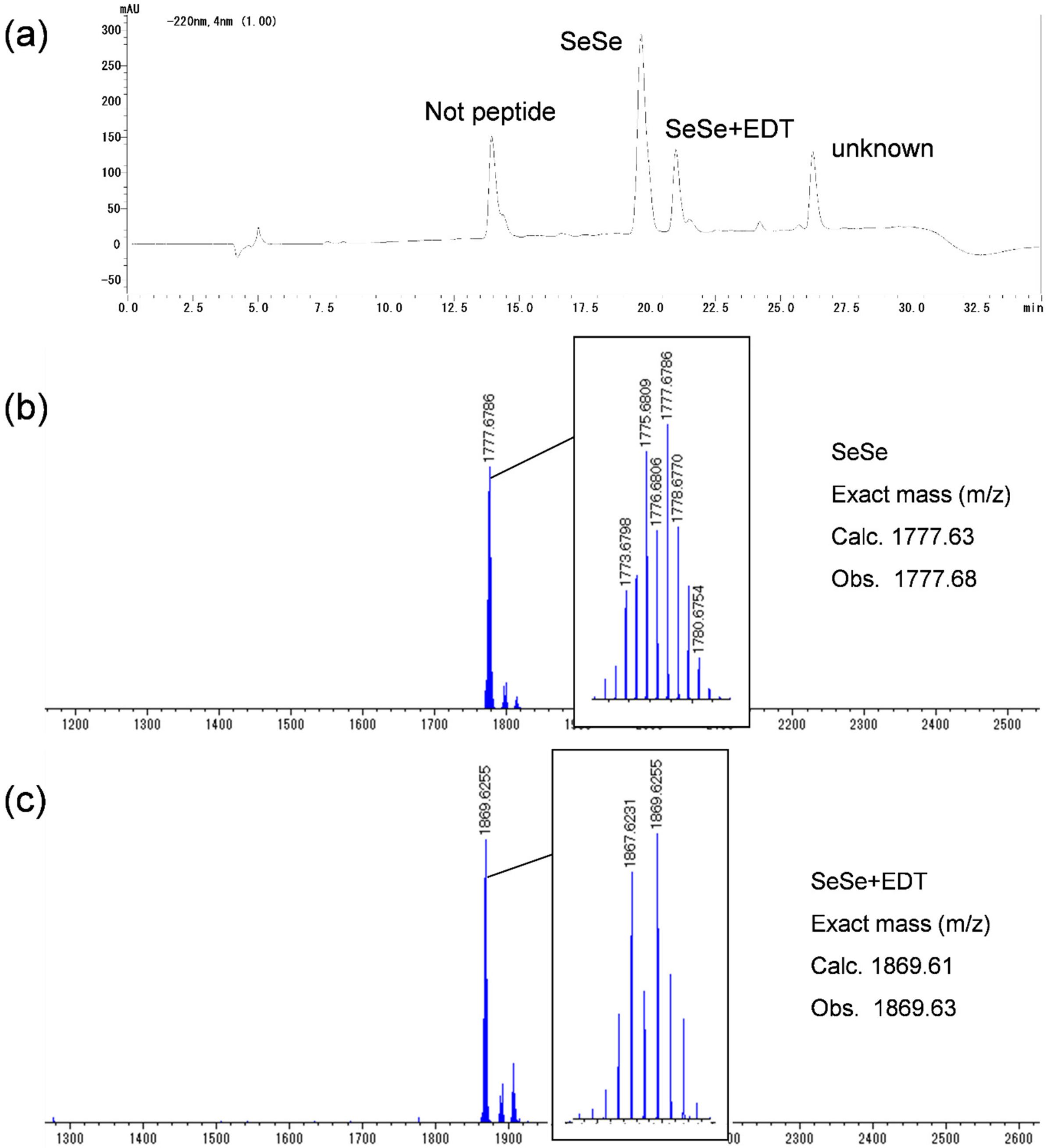

Figure S4. HPLC chart and MALDI-TOF-MS spectra for (H-UAAAAHGEL-OH)$_2$ cleaved from H-UAAAAHGEL-PAM (**5d**). (a) HPLC chart of the cleaved peptide mixture. (b) MALDI-TOF-MS spectrum for the SeSe peak. (c) MALDI-TOF-MS spectrum for the SeSe+EDT peak.

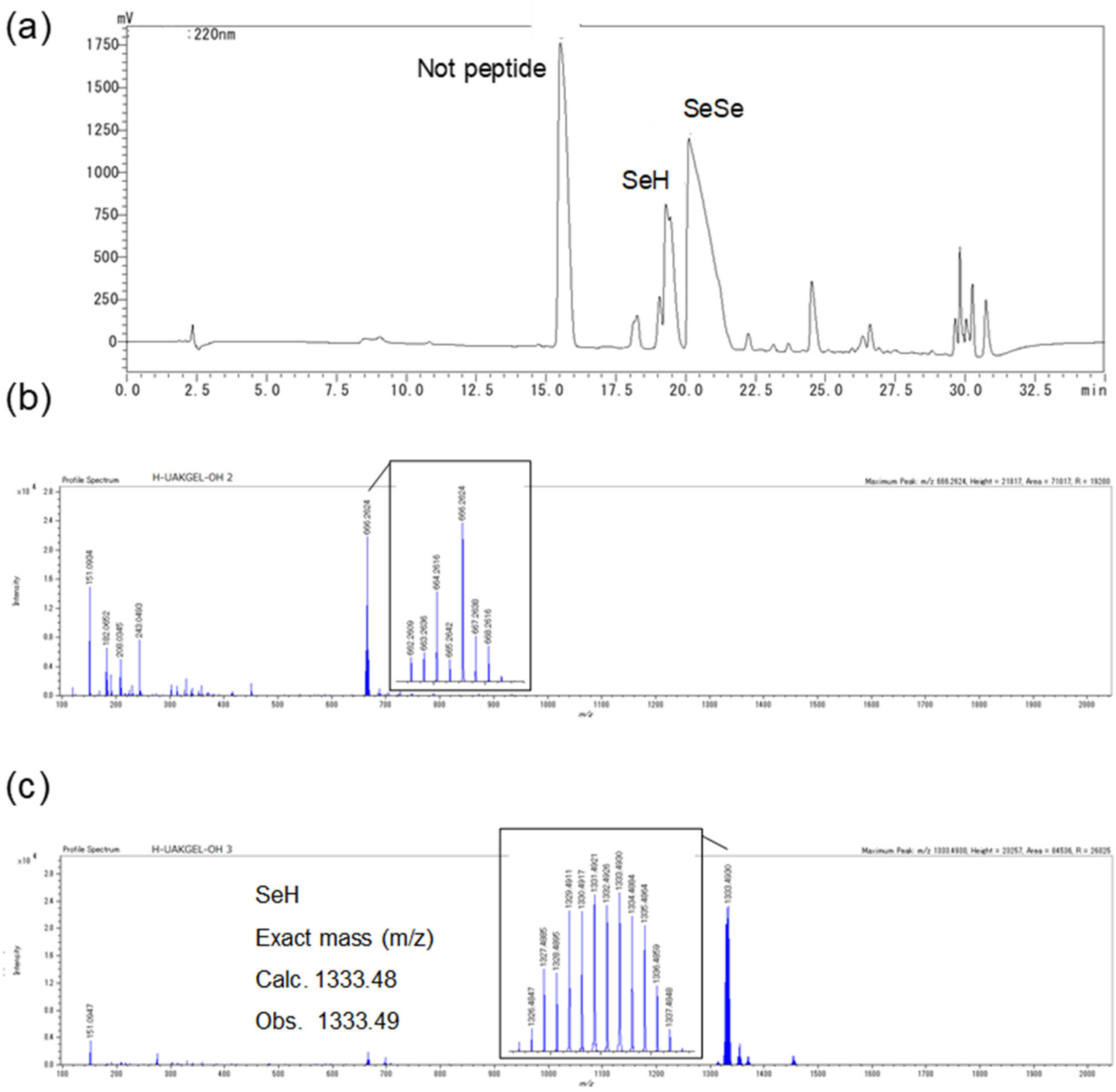

Figure S5. HPLC chart and MALDI-TOF-MS spectra for $(H\text{-}UAKGEL\text{-}OH)_2$ cleaved from H-UAKGEL-PAM (**5e**). (a) HPLC chart of the cleaved peptide mixture. (b) MALDI-TOF-MS spectrum for the SeH peak. (c) MALDI-TOF-MS spectrum for the SeSe peak.

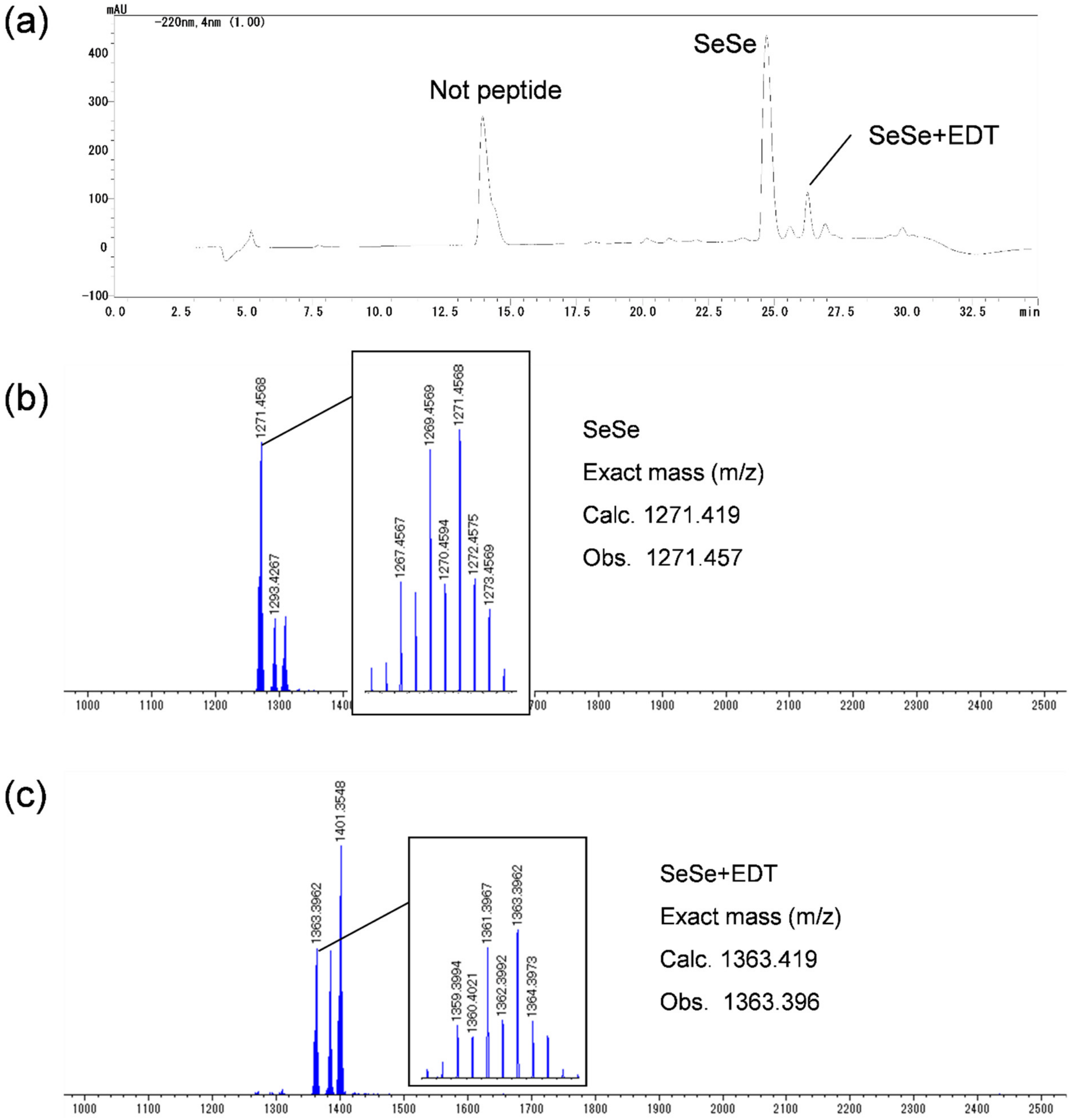

Figure S6. HPLC chart and MALDI-TOF-MS spectra for (H-UAPGEL-OH)$_2$ cleaved from H-UAPGEL-PAM (**5f**). (a) HPLC chart of the cleaved peptide mixture. (b) MALDI-TOF-MS spectrum for the SeSe peak. (c) MALDI-TOF-MS spectrum for the SeSe+EDT peak.

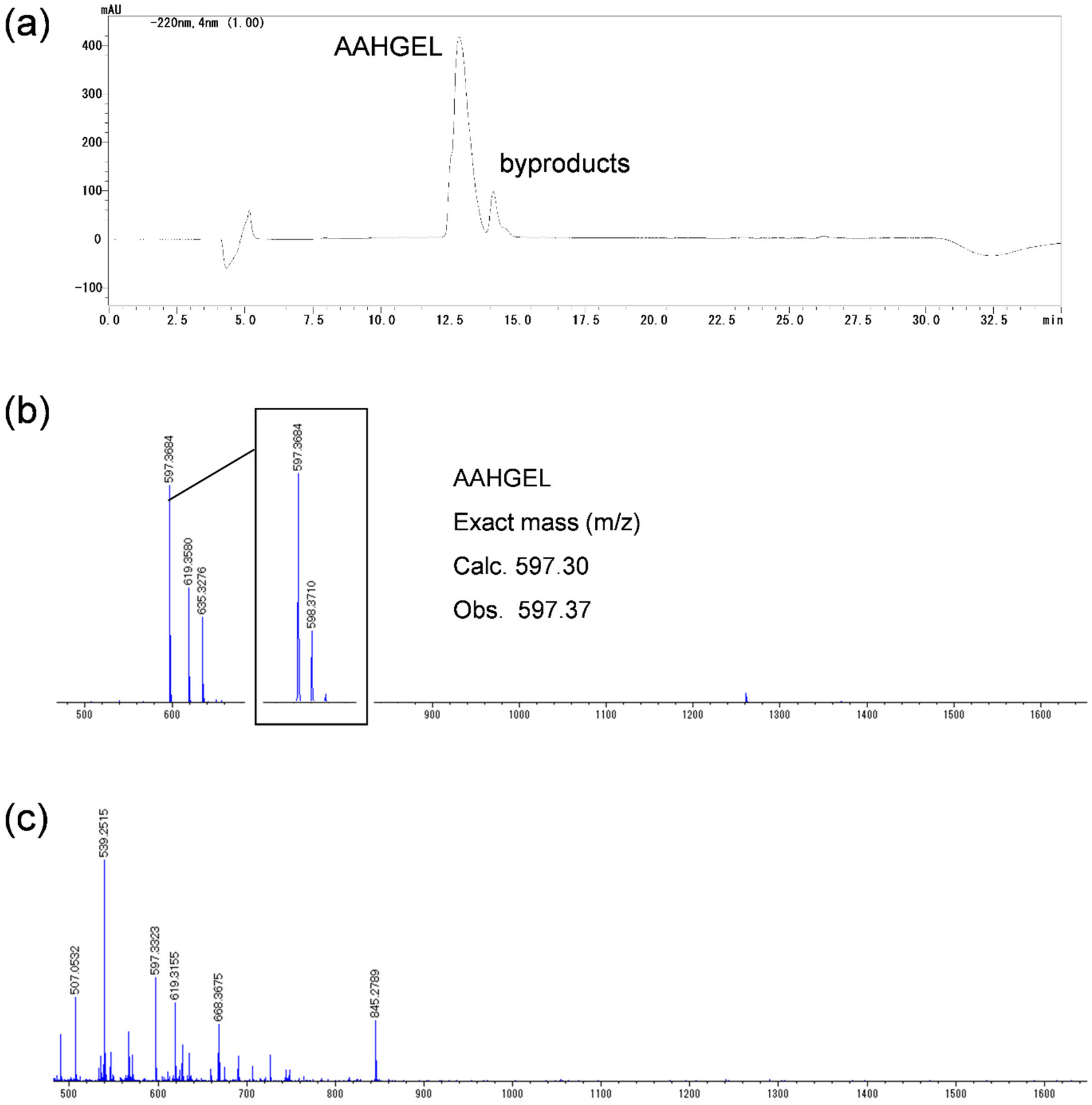

Figure S7. HPLC chart and MALDI-TOF-MS spectra for H-AAHGEL-OH cleaved from H-AAHGEL-PAM (**5g**). (a) HPLC chart of the cleaved peptide mixture. (b) MALDI-TOF-MS spectrum for the AAGEL peak. (c) MALDI-TOF-MS spectrum for the byproducts peak.

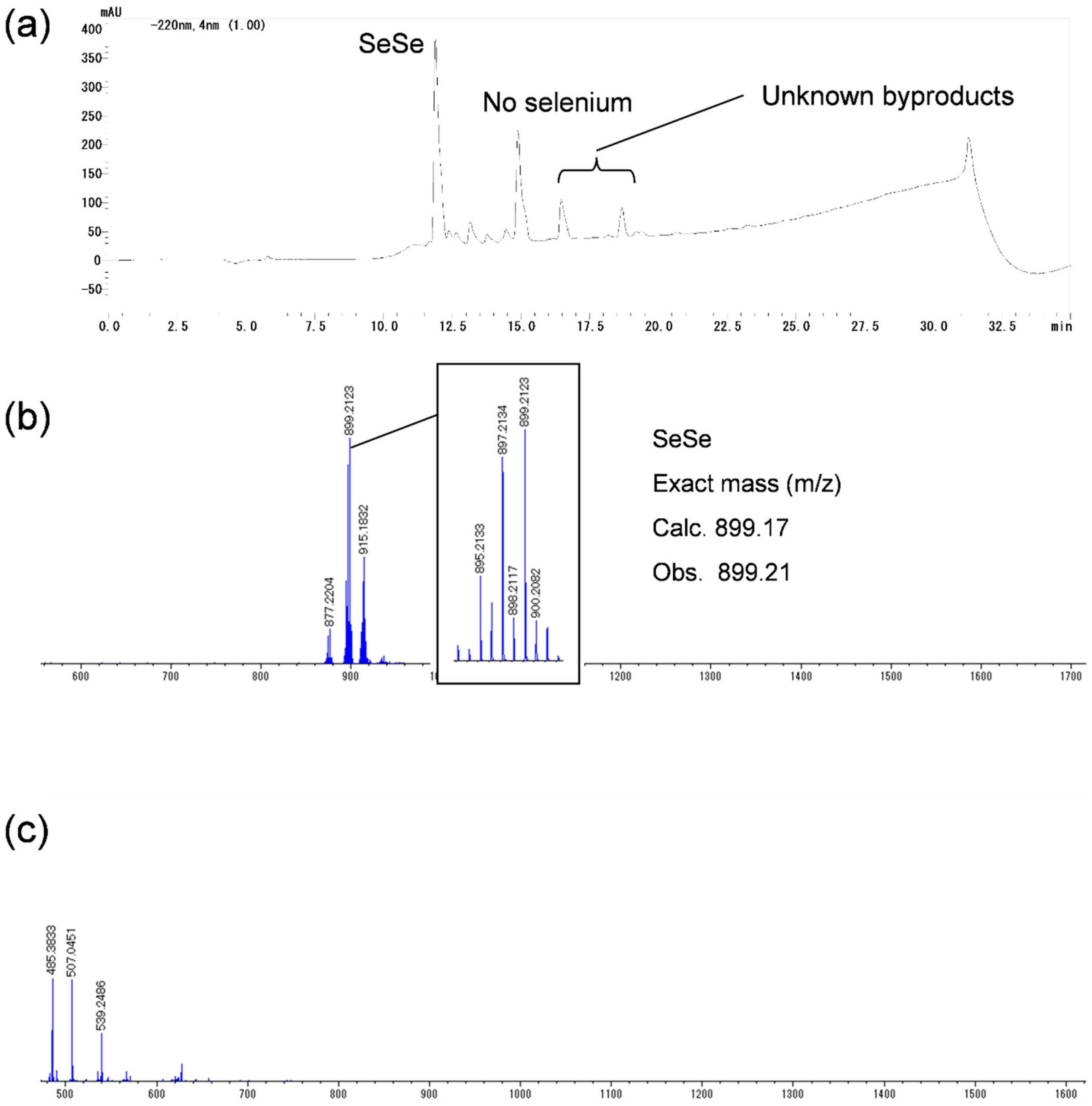

Figure S8. HPLC chart and MALDI-TOF-MS spectra for (H-UAAAG-OH)$_2$ cleaved from H-U*AAAG-PAM, a synthetic precursor of **6a**. (a) HPLC chart of the cleaved peptide mixture. (b) MALDI-TOF-MS spectrum for the SeSe peak. (c) MALDI-TOF-MS spectrum for the no-selenium peak.

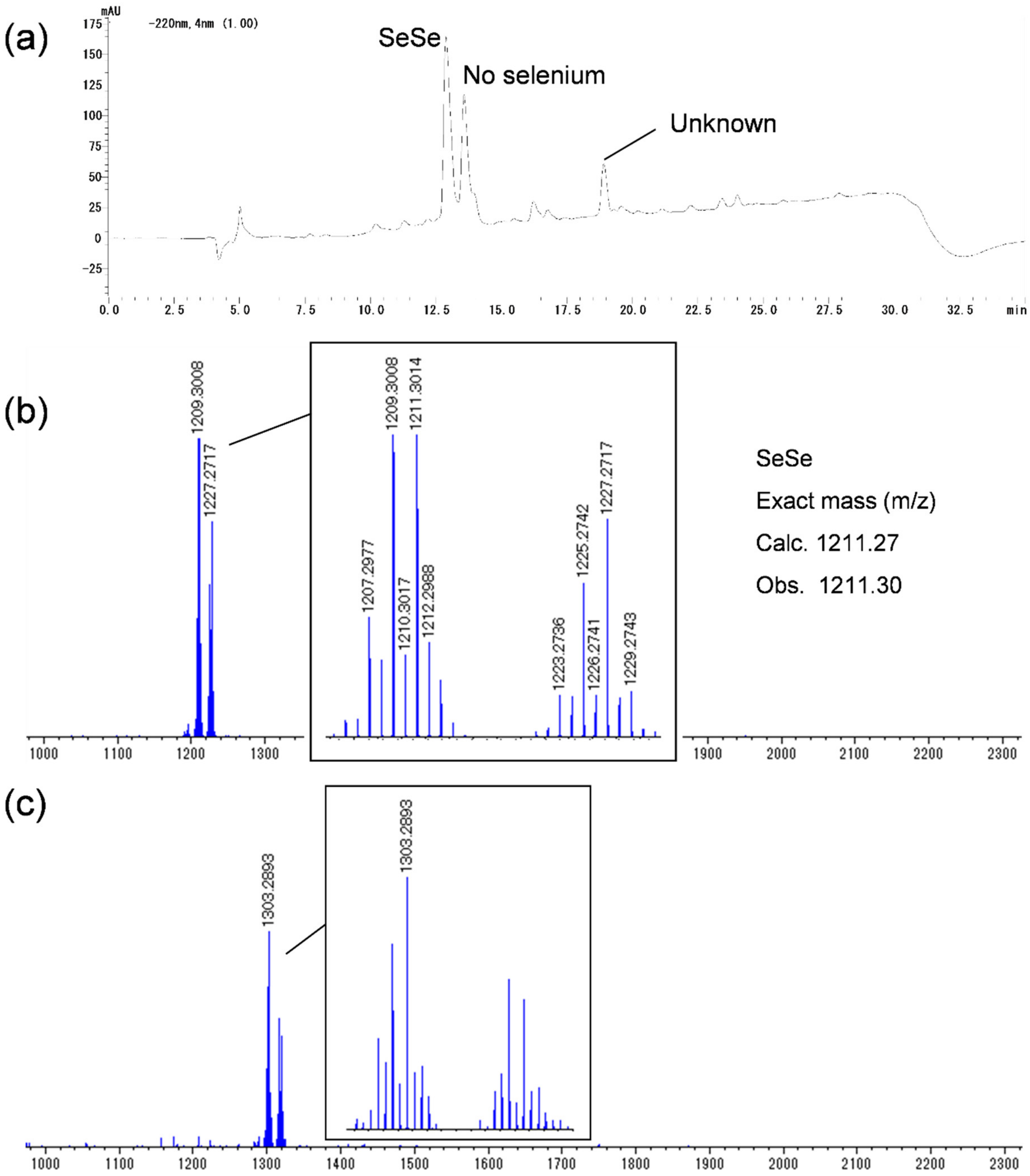

Figure S9. HPLC chart and MALDI-TOF-MS spectra for $(Ac\text{-}UAAAGGG\text{-}OH)_2$ cleaved from Ac-U*AAAGGG-PAM (**6b**). (a) HPLC chart of the cleaved peptide mixture. (b) MALDI-TOF-MS spectrum for the SeSe peak. (c) MALDI-TOF-MS spectrum for the unknown peak.

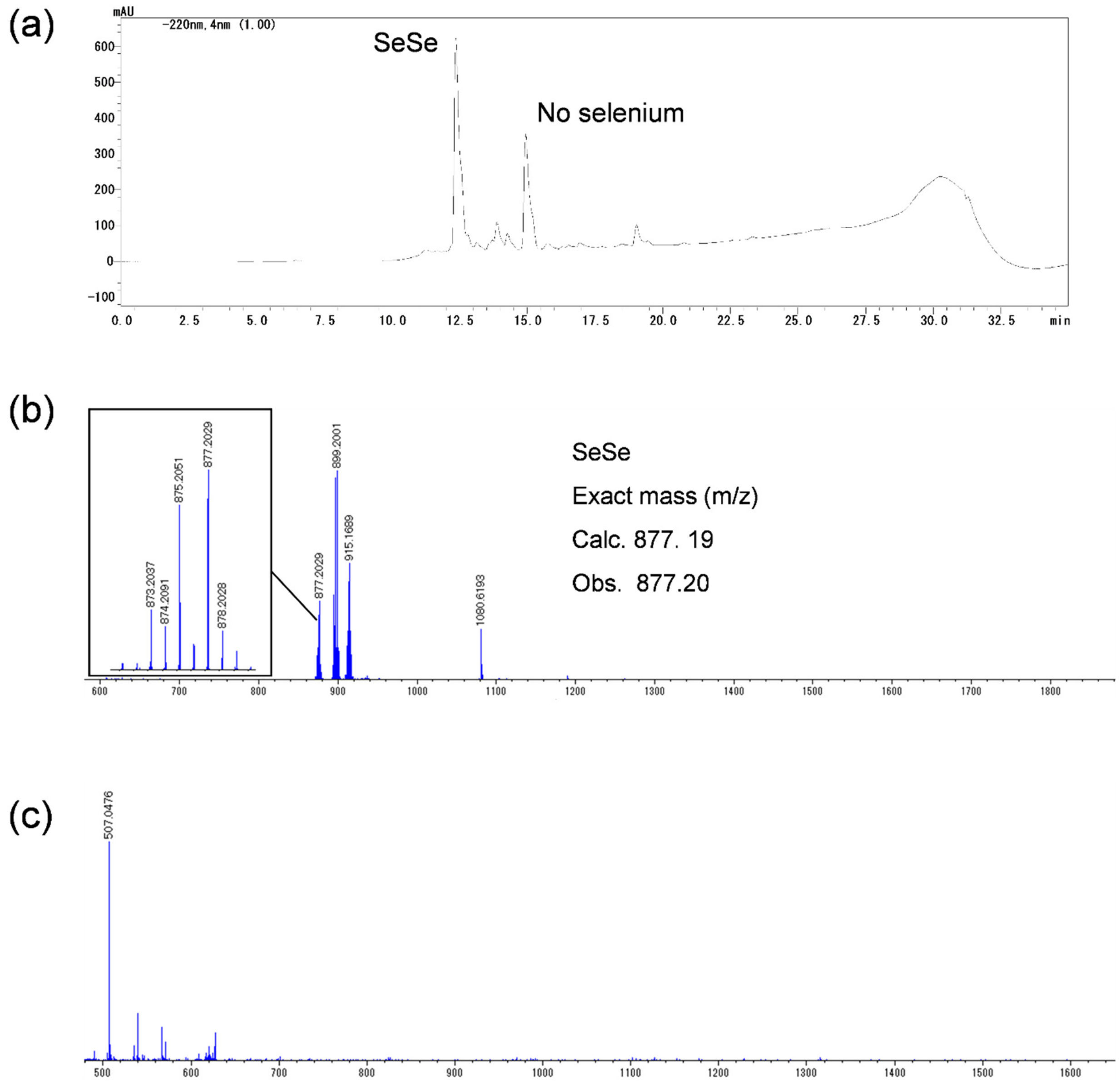

Figure S10. HPLC chart and MALDI-TOF-MS spectra for (H-AUAAG-OH)$_2$ cleaved from H-AU*AAG-PAM, a synthetic precursor of **6d**. (a) HPLC chart of the cleaved peptide mixture. (b) MALDI-TOF-MS spectrum for the SeSe peak. (c) MALDI-TOF-MS spectrum for the no-selenium peak.

2. Experimental data of the GPx-like peroxidase activity assays by the UV method.

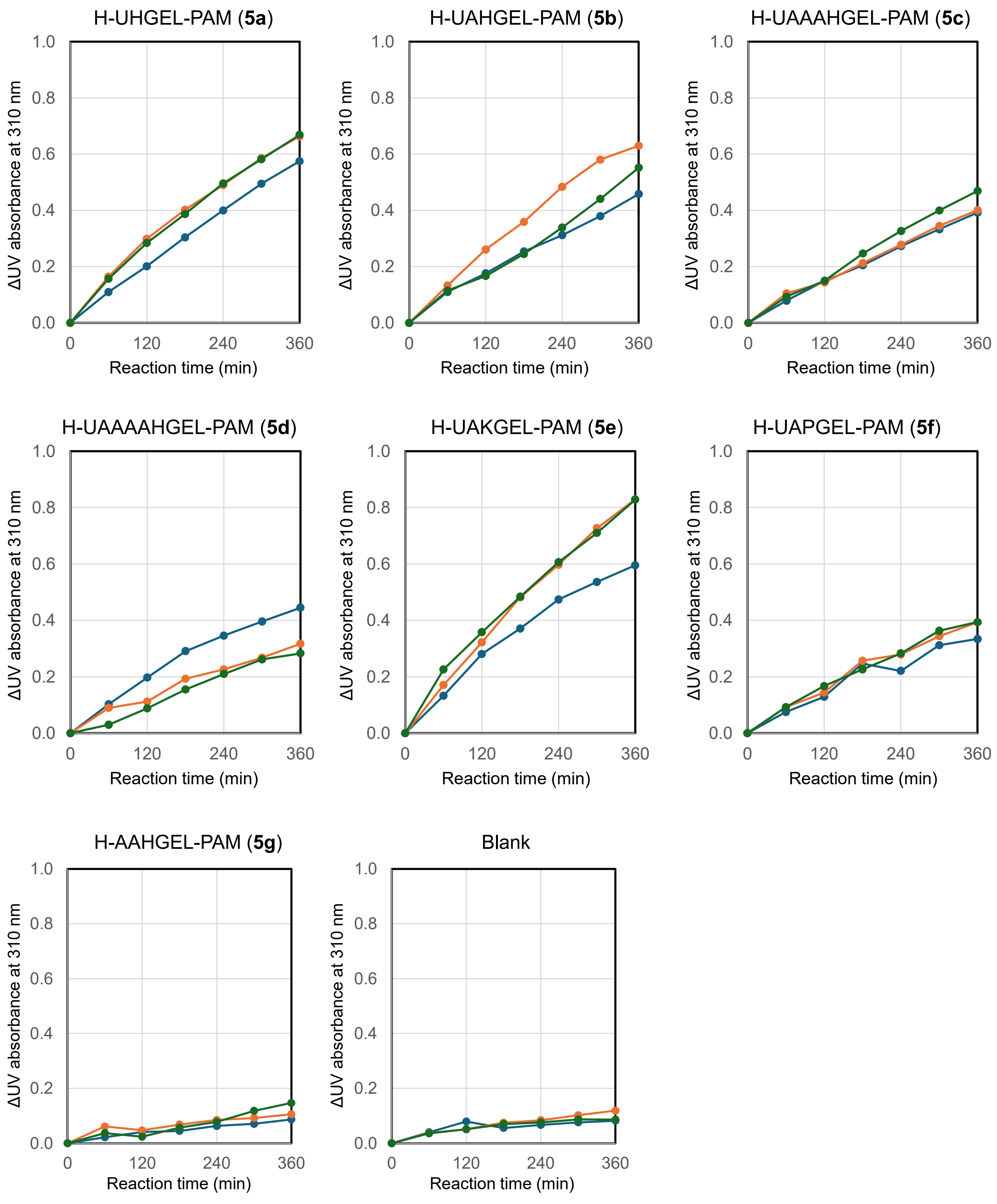

Figure S11. GPx-like peroxidase activity assays for **5a-g** by the UV method.

3. Experimental data of the GPx-like peroxidase activity assays by ^{1}H NMR.

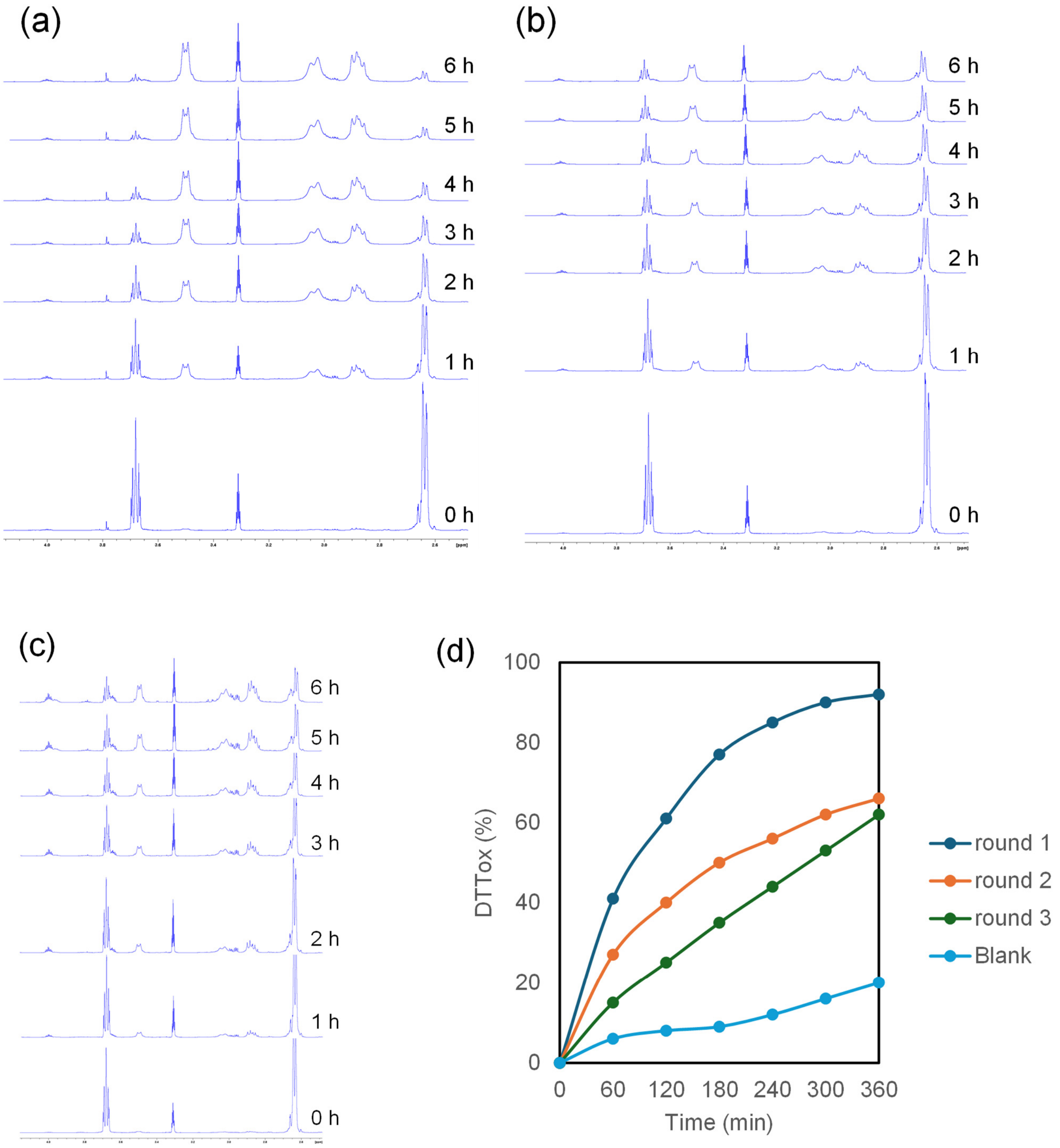

Figure S12. GPx-like peroxidase activity assays for H-UAKGEL-PAM (**5e**) by ^{1}H NMR. (a) Round 1. (b) Round 2. (c) Round 3. (d) Populations of DTT^{ox} produced in the reaction between H_2O_2 and DTT^{red} in CD_3OD at 300 K.

4. Molecular structures of H-UAKGEL-OH obtained by REMC/SAAP3D simulation and the subsequent clustering analysis.

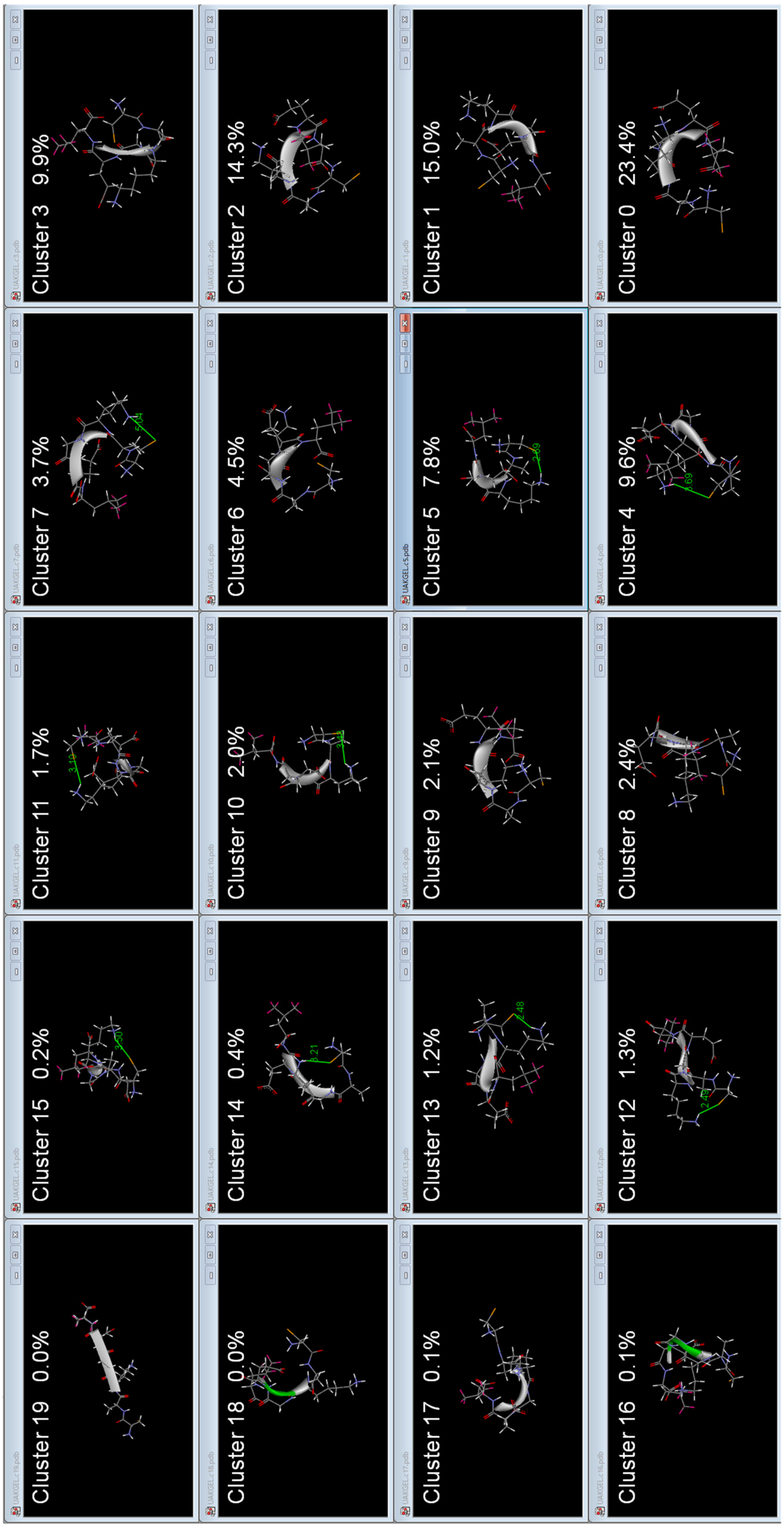

Figure S13. Molecular structures of H-UAKGEL-OH obtained by REMC/SAAP3D simulation.

5. ^{1}H NMR spectra for cyclized products 8a, 8b, 8d, and 8e.

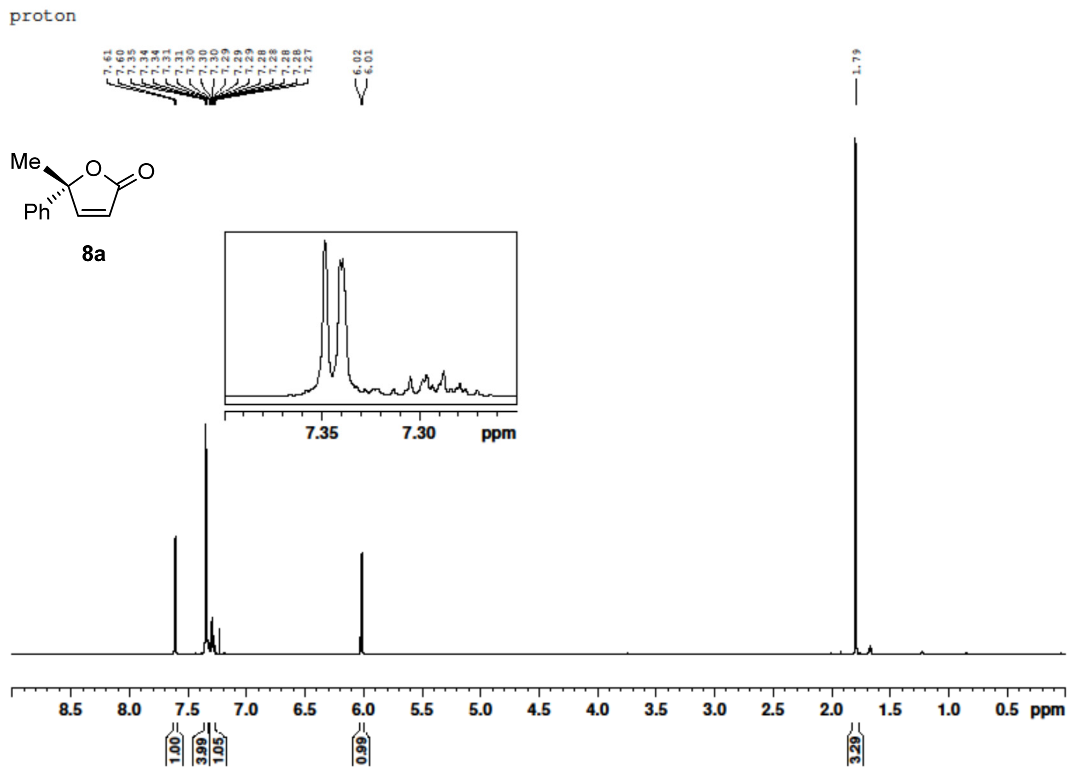

Figure S14. 500 MHz ^{1}H NMR spectrum for **8a** in $CDCl_3$.

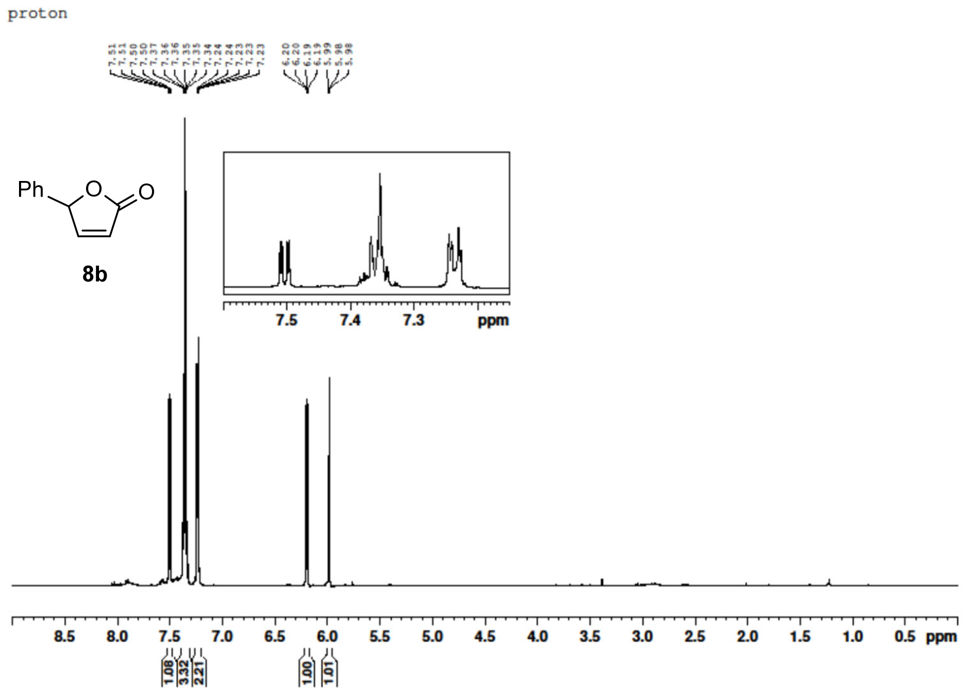

Figure S15. 500 MHz ^{1}H NMR spectrum for **8b** in $CDCl_3$.

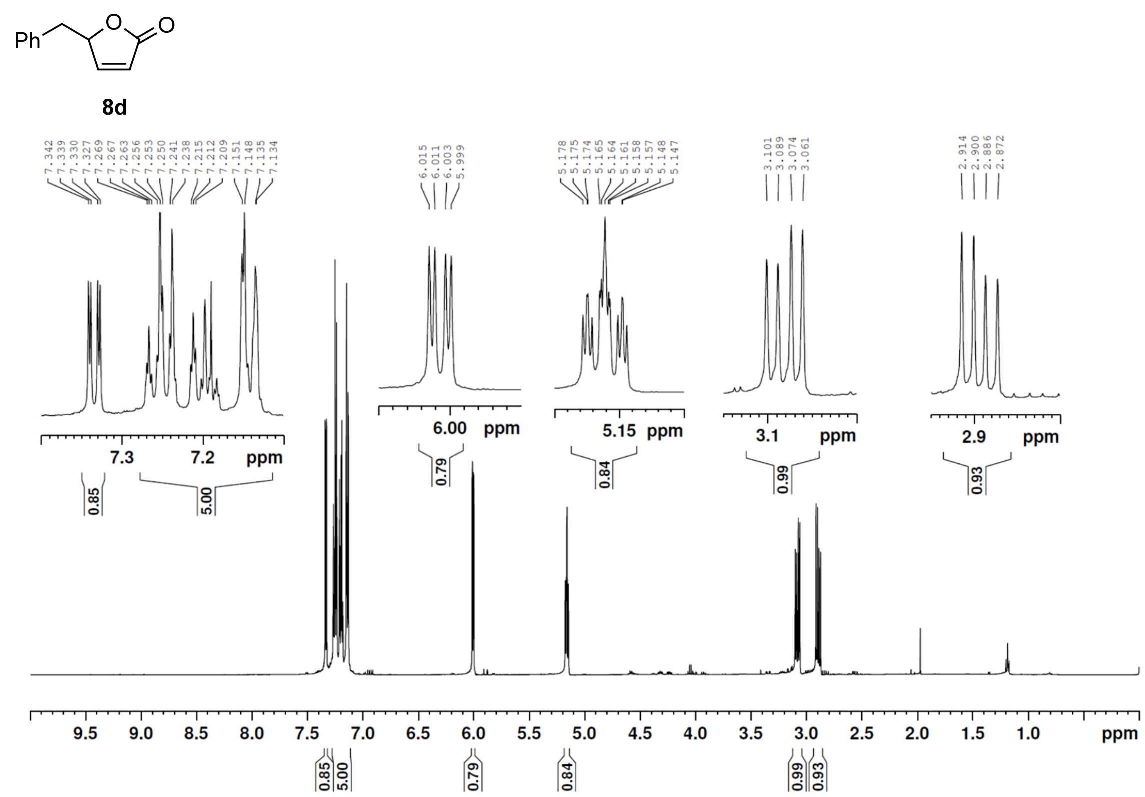

8d

Figure S16. 500 MHz ^{1}H NMR spectrum for **8d** in $CDCl_3$.

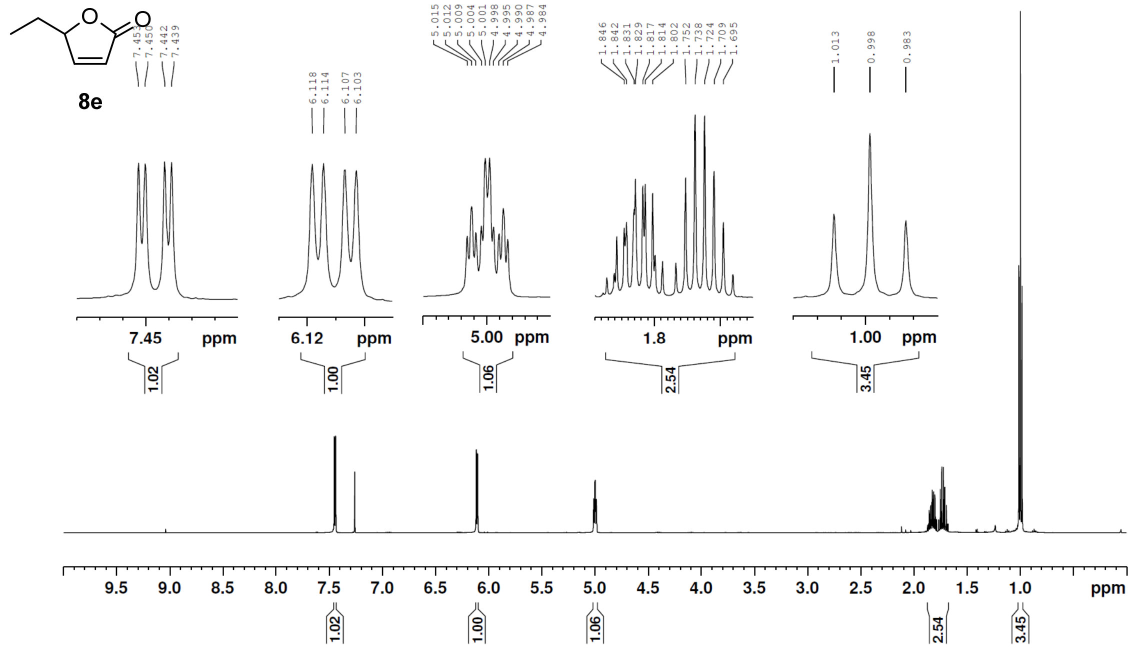

8e

Figure S17. 500 MHz ^{1}H NMR spectrum for **8e** in $CDCl_3$.

6. Determination of the enantiomeric excess for cyclized product 8a.

A reference sample of **8a** was synthesized according to the procedure reported by Tan et al. (Tan, C.K.; Er, J.C.; Yeung, Y.-Y. *Tetrahedron Lett.* **2014**, *55*, 1243–1246) (Scheme 1). The enantiomeric excess (e.e. = (*R*–*S*)/(*R*+*S*)) of the obtained sample was determined to be 28 % e.e. by RP-HPLC using a chiral column (Figure S18).

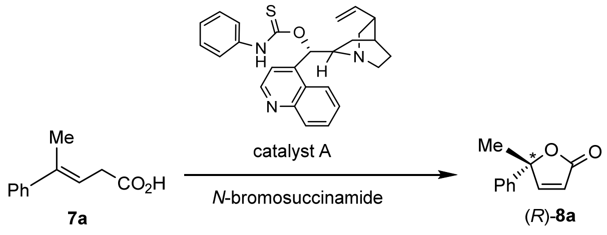

Scheme S1

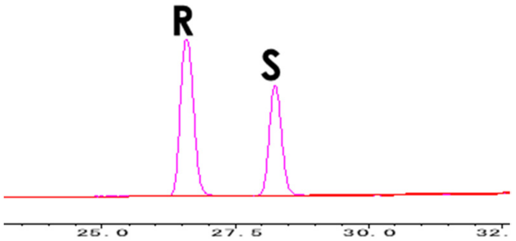

Figure S18. HPLC analysis for **8a** synthesized according to Scheme 1. HPLC analysis conditions: Column: Chiralcel OD-RH (0.46 x 15 cm, Daicel Chemical), Solvent gradient: 20–50% MeCN in water with 0.1% TFA in 25 min, Flow rate: 0.4 mL/min, Column temperature: 35ºC, UV wavelength: 215 nm.

Based on the results, the enantiomeric excesses of **8a**, which were obtained by using on-resin catalyst **6** under various conditions, were determined as shown in Table S1.

Table S1. Enantiomeric excesses of **8a**.

Entry	Substrate	Se-resin	Solvent	Conditions	Round	Yields of 8a	% e.e.
2	**7a**	**6b**	MeCN (10 mL)	60°C, 18h	1	25 %	6
3	**7a**	**6a**	MeCN (1 mL)	60°C, 18h	1	79 %	1
					2	73 %	2
					3	27 %	6
					4	0 %	
4	**7a**	**6b**	MeCN (1 mL)	60°C, 18h	1	33 %	14
					2	51 %	2
					3	14 %	6
					4	0 %	

Conditions of entries were the same as those shown in Table 2 in the main text.

(*R*)-isomer was obtained as a major product.

7. The second-order rate constants (k_2) for 5a-g in comparison with those reported for other selenopeptides.

Table S2. The second-order rate constants (k_2) in comparison with the initial rates (v_0) reported for various selenopeptides.

catalysts	Amino acid sequence	Second-order rate constants (k_2) (μM^{-1} min^{-1})	Relative activity against blank	Conditions	References
**5a**	H-UHGEL-PAM	0.288 ± 0.041	10.7	Cat **5**: 10 mg<br>$[DTT^{red}] = [H_2O_2]$ = 10 mM<br>in CH_3OH at r.t.	This work
**5b**	H-UAHGEL-PAM	0.217 ± 0.056	8.0		
**5c**	H-UAAAHGEL-PAM	0.142 ± 0.016	5.3		
**5d**	H-UAAAAHGEL-PAM	0.117 ± 0.035	4.3		
**5e**	H-UAKGEL-PAM	0.430 ±0.114	15.9		
**5f**	H-UAPGEL-PAM	0.127 ± 0.011	4.7		
**5g**	H-AAHGEL-PAM	0.029 ± 0.005	1.1		
blank		0.027 ± 0.003	1.0		

Selenopeptides	Initial rates (v_0) (μM min^{-1})	Relative initial rates	Conditions	
(H-γEUG-OH)$_2$ (selenoglutathione)	2000 ± 100	18.2	[catalyst] = 20 μM<br>$[DTT^{red}]$ = 10 mM<br>$[H_2O_2]$ = 2 mM<br>in pH 7.0 phosphate buffer at 25°C	[15]
(H-LUG-OH)$_2$	1500 ± 100	13.6		
(H-SLUGT-OH)$_2$	1600 ± 100	14.5		
(H-U-OH)$_2$ (selenocystine)	1400 ± 100	12.7		
blank	110 ± 10	1.0		
H-CUGEGE-OH		2.42	[catalyst] = 50 μM<br>[GSH] = 4.0 mM<br>$[H_2O_2]$ = 0.25 mM<br>[NADPH] = 0.30 mM<br>[glutathione reductase] = 13 units/mL<br>in pH 7.4 phosphate buffer at 25°C	[14]
blank		1.0		
(H-GAAUAAG-OH)$_2$	23 ± 2	2.3	[catalyst] = 50 μM<br>[GSH] = 4.0 mM<br>$[H_2O_2]$ = 0.25 mM<br>[NADPH] = 0.30 mM<br>[glutathione reductase] = 4 units/mL<br>in pH 7.4 phosphate buffer at 25°C	[16]
(H-GAAUAWG-OH)$_2$	19 ± 1	1.9		
(H-GQAUAAG-OH)$_2$	33 ± 5	3.3		
(H-GQAUAWG-OH)$_2$	37 ± 3	3.7		
blank	10 ± 2	1.0		
[cyclic-KQGUGWGN-]$_2$	89 ± 8	5.2	[catalyst] = 50 μM<br>[GSH] = 4.0 mM<br>$[H_2O_2]$ = 0.25 mM<br>[NADPH] = 0.30 mM<br>[glutathione reductase] = 4 units/mL<br>in pH 7.4 phosphate buffer at 25°C	[17]
[cyclic-KQGUGAGN-]$_2$	84 ± 4	4.9		
[cyclic-KQGUGWGA-]$_2$	72 ± 3	4.2		
[cyclic-KAGUGWGN-]$_2$	38 ± 4	2.2		
blank	17 ± 1	1.0		
